# Impact of framework material, cantilever design, and wing configuration on stress distribution in patient specific additively manufactured subperiosteal jaw implants: a 3D finite element analysis

**DOI:** 10.1186/s12903-025-07214-5

**Published:** 2025-11-21

**Authors:** Gokhan Canko, Ozge Doganay Ozyilmaz

**Affiliations:** 1 Department of Oral and Maxillofacial Surgery, Institute of Health Sciences, Bezmialem Vakıf University, Istanbul, Türkiye; 2 Faculty of Dentistry, Department of Oral and Maxillofacial Surgery, Bezmialem Vakif University, Adnan Menderes Vatan Bulvarı, Vatan Caddesi, Istanbul, 34093 Türkiye

**Keywords:** Additively manufactured subperiosteal jaw implants, Polyetheretherketone, Cobalt-chromium, Cantilever, Wing design, Prosthetic displacement, Finite element analysis

## Abstract

**Statement of problem:**

Recent advancements in digital technology have revolutionized implant dentistry, particularly with additively manufactured subperiosteal jaw implants (AMSJIs). These implants allow patient-specific designs that adapt to anatomical requirements. However, optimizing stress distribution remains a challenge.

**Purpose:**

This study evaluated the stress distribution in AMSJIs and surrounding bone by analyzing different framework materials (PEEK and Co-Cr), anterior wing designs (I- and Y-shaped), and cantilever extensions using three-dimensional finite element analysis.

**Methods:**

A model was created from a patient with an atrophic, edentulous maxilla. Biomechanical evaluation of eight maxillary implant scenarios was performed under a 200 N force applied at a 45° oblique angle. Stress distribution in the bone, implants, screws, and prosthetic frameworks, as well as prosthetic displacement, was analyzed.

**Results:**

The lowest implant stress (444.5 MPa) was observed in the Co-Cr group without a cantilever using an I-shaped design, whereas the highest stress (623.0 MPa) occurred in the Co-Cr group with a cantilever using a Y-shaped design. Prosthetic displacement was greater in cantilevered groups, with PEEK exhibiting more displacement than Co-Cr.

**Conclusions:**

The optimal stress distribution was achieved with the I-shaped design without a cantilever, using Co-Cr. Stress levels were significantly influenced by framework material, wing design, and cantilever presence, underscoring the importance of design and material selection.

**Clinical significance:**

While stress remained within physiological limits in all cases, avoiding cantilevers and selecting a rigid material can optimize Y-shaped designs. PEEK demonstrated favorable properties in cantilevered designs, but its long-term effects on soft tissue and implants warrant further clinical trials.

## Introduction

The rehabilitation of atrophic jaws remains one of the most complex challenges in maxillofacial clinical practice. Treatment options for severe bone atrophy include bone grafting, the All-on-4 technique, quadzygoma implants, or a combination of these approaches. However, bone grafting carries risks of complications, including nerve injury, infection, and oroantral fistula formation, while zygomatic implants require advanced surgical expertise to avoid maxillary sinus perforation.

The widespread adoption of three-dimensional (3D) tomography in dentistry, combined with technological advancements, has aimed to overcome these limitations. In cases where bone augmentation procedures or other techniques are not feasible or have failed, the additively manufactured subperiosteal jaw implant (AMSJI) has re-emerged as a valuable clinical alternative for patients with severe maxillary atrophy [1]. This innovative subperiosteal implant concept leverages computer-aided design and manufacturing (CAD/CAM) alongside laser melting technologies, enabling the development of implants with diverse shapes and geometries. By facilitating the production of highly customized implants that achieve an optimal fit to the patient’s anatomy, this approach significantly enhances the potential for clinical success [1–4].

AMSJIs are custom-designed based on an individual’s anatomical structure, bone thickness and volume, surgical factors, and load-bearing capacity. This individualized approach is crucial in achieving optimal functional integration and minimizing complications [1].

Occlusal forces applied to the implant create stress in the peri-implant region, influenced by factors such as cantilever presence, implant design, and prosthetic material properties. Although a prosthesis without a cantilever provides the optimal stress distribution, cantilever-extended prostheses may be necessary when bone anatomy limits the placement of a sufficient number of implants, ensuring biomechanical stability [5].

Prosthetic framework materials are critical to the biomechanical success of both the prosthesis and implants. Framework material hardness significantly impacts force distribution, and research suggests that microstrains between 50 and 1500 μm indicate bone stability with no significant volume changes [6–8]. Bone resorption may occur due to stress shielding, which reduces force transmission to the bone or as a result of pathological overload [9–11].

Metal frameworks typically offer excellent mechanical properties for prosthetic designs. However, concerns about their potential to contribute to bone resorption have driven increased research into materials with an elastic modulus closer to that of bone. Recently, polyetheretherketone (PEEK) has been introduced into clinical practice due to its acceptable mechanical properties, offering enhanced stress distribution and a reduced risk of biomechanical complications [12, 13].

Finite element analysis (FEA) provides a powerful tool to investigate the biomechanical performance of implant systems by transforming three-dimensional anatomical structures into digital models. This approach enables detailed assessment of stress distribution and concentration points within implants, screws, and surrounding bone, thereby identifying potential areas of mechanical risk. The integration of advanced digital technologies with FEA offers the opportunity to preoperatively simulate complex scenarios and optimize implant design before clinical application.

The present study employs FEA to evaluate the influence of framework material selection (PEEK and Cobalt–chromium), anterior wing geometry (I- and Y-shaped designs), and the presence or absence of cantilever extensions on stress distribution. In contrast to previous investigations that primarily examined conventional I-shaped anterior wings under ideal anatomical conditions [1, 14–18, 19, 20, 21, 22], this work specifically focuses on clinical scenarios characterized by bone insufficiency, deformation, or anatomical variation, where lateral screw placement adjacent to the nasal cavity is impractical By introducing modifications to the standard I-shaped AMSJI configuration and systematically comparing these alternatives under functional loading, the study provides a novel perspective not previously addressed in the literature.

## Materials and methods

This study was approved by the Ethics Committee of Bezmialem Vakif University (Decision No: 2022/381) and supported by the Scientific Research Projects Coordination Unit of Bezmialem Vakif University (Project No: 2023/0624).

A 3D model of an atrophic maxilla was generated from computed tomography (CT) data of an adult patient. The dataset was initially saved in STL format and subsequently converted to Digital Imaging and Communications in Medicine (DICOM) format using 3D-Doctor software (Able Software Corp.). Cortical and cancellous bone structures were reconstructed and processed into finite element analysis (FEA) models. The bone model was created and exported as an STL file using 3D Slicer, while reverse engineering and CAD operations were performed in ANSYS SpaceClaim. Solid model adaptation for analysis, mesh optimization, and FEA simulations were conducted in ANSYS Workbench, with solutions obtained using the LS-DYNA solver. Force-loading simulations involving prosthetic components, subperiosteal implant screws, and bone were modeled using the Boolean method. Accurate spatial positioning of all components was achieved in Rhinoceros 4.0, and a detailed 3D mesh was generated in VRMesh Studio (VirtualGrid Inc.). All mesh generation, transformation to solid mesh, and stress analyses were performed on HP workstations equipped with Intel Xeon E-2286 processors (2.40 GHz) and 64 GB ECC memory.

**Fig. 1:**
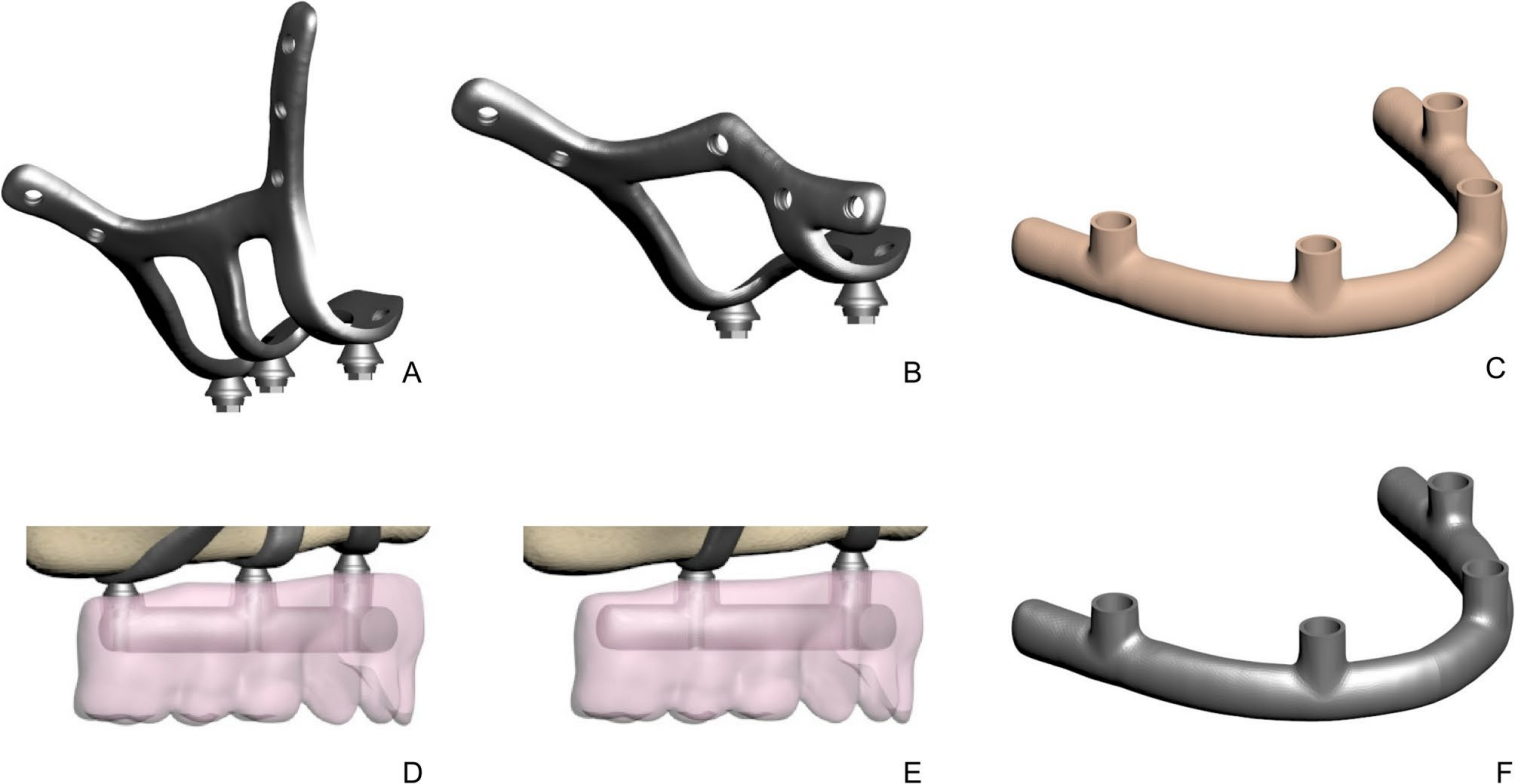
Subperisteal implant components. A, I-shaped design without cantilever. B, Y-shaped design with cantilever. C, Peek infrastructure. D, Prosthesis and infrastructure without cantilever E, Prosthesis and infrastructure with cantilever. F, Co-cr infrastructure

The AMSJI design consisted of three components: a left and right AMSJI subunits and an intraoral connector. Figure 1A and B illustrate two variations of the AMSJI design, featuring either an I-shaped or Y-shaped anterior wing. The zygomatic (posterior) and nasal (anterior) wings were connected to the basal looped frame, with two or three arms (Fig. 1D and E), depending on the design, linking the basal looped frame to the abutments. The wings were designed with holes for osteosynthesis screw placement. To optimize load distribution, a prosthetic framework was designed with or without a distal cantilever (Table 1). Tooth sizes for the superstructure were selected based on Wheeler’s Dental Anatomy Atlas. A comprehensive list of all components used in the study is provided in Table 2. The subperiosteal implant and the abutment were designed as a single, unified component. The PEEK and Co-Cr frameworks were directly screwed to the transepithelial abutments without any additional interface. In all analyses, the contacting components were defined as’’bonded’’.

**Table 1:** AMSJI Groups, nodes and elements

Group	Anterior Wing Design	Framework	Nodes	Elements
1	I-shaped	Co-Cr without cantilever	614,346	2,403,961
2	I-shaped	Co-Cr with cantilever	558,933	2,185,636
3	I-shaped	PEEK without cantilever	614,346	2,403,961
4	I-shaped	PEEK with cantilever	558,933	2,185,636
5	Y-shaped	Co-Cr without cantilever	585,227	2,283,600
6	Y-shaped	Co-Cr with cantilever	532,796	2,078,377
7	Y-shaped	PEEK without cantilever	585,227	2,283,600
8	Y-shaped	PEEK with cantilever	532,796	2,078,377

*Co-Cr* Cobalt-chromium, *PEEK* Polyetheretherketone

**Table 2:** Details of all components of subperiosteal implant and screws

	Diameter/Thickness	Length/width
Subperiosteal Implant **(**Ti Grade 5)	1.3 mm in thickness	4 mm in width
Occlusal screw	2.2 mm in diameter	3.7 in length
Fixation screw in piriform apertura (#1 and #2) **(**Ti Grade 4)	2 mm in diameter	5 mm in length
Fixation screw in zygomatic bone (#3 and #4) **(**Ti Grade 4)	2 mm in diameter	10 mm in length
Fixation screw in palatinal region (#5) **(**Ti Grade 4)	2 mm in diameter	5 mm in length
Abutment	4,1 mm in diameter	1.5 mm
PEEK Framework	5 mm in thickness	5 mm in width
Co-Cr Framework	5 mm in thickness	5 mm in width
Fixed prosthesis **(**Acrylic resin)	15 mm in thickness	12 teeth
Cantilever	-	10 mm in length

*Co-Cr* Cobalt-chromium, *PEEK* Polyetheretherketone, *Ti* titanium

**Fig. 2:**
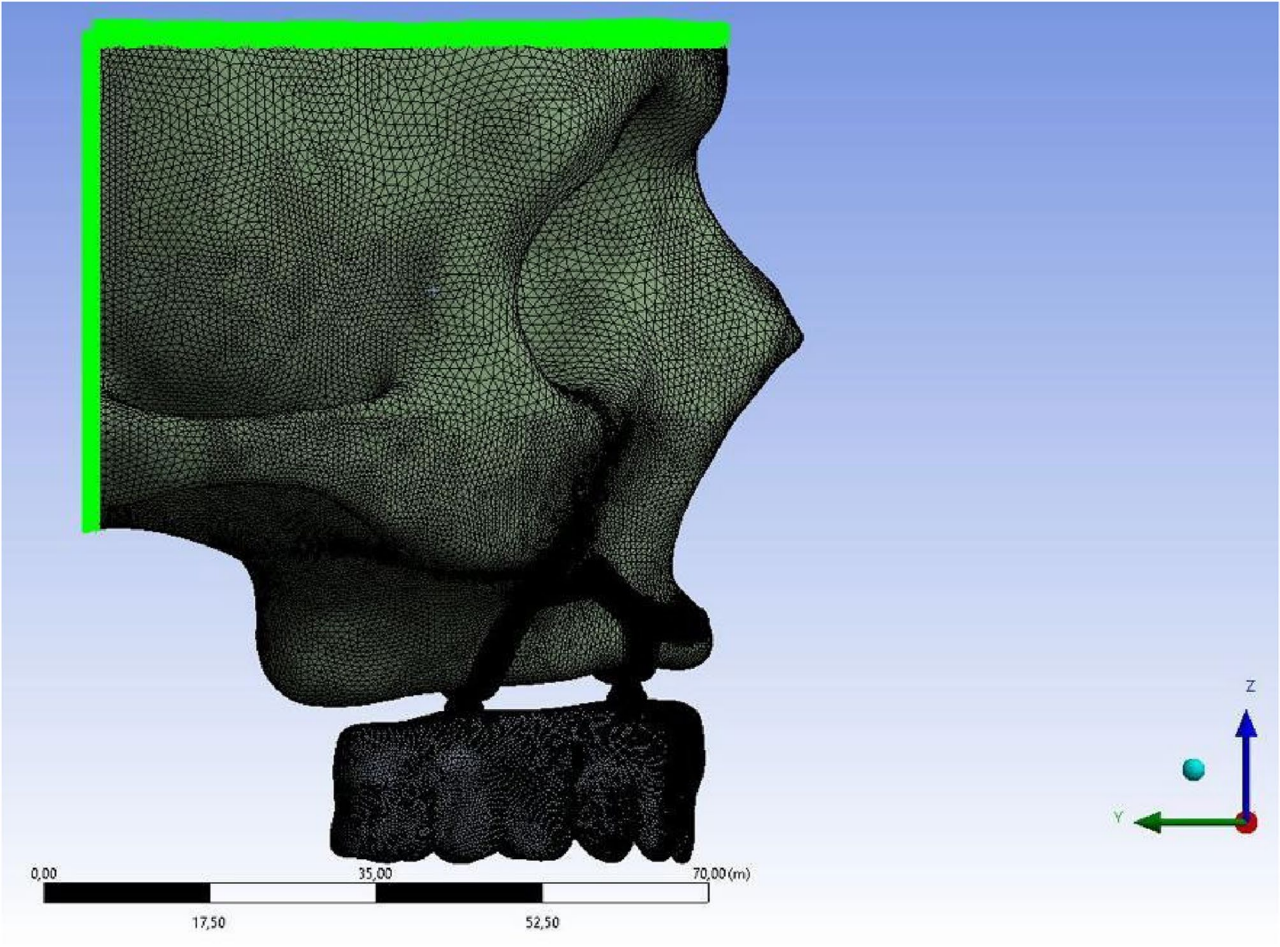
Boundary conditions

The model was fixed by restricting all degrees of freedom at nodal points located in the superior and posterior regions of the bone, effectively preventing movement along any of the three axes. Additionally, boundary conditions were symmetrically applied in the Y-Z plane relative to the X-axis for all components in the model (Fig. 2).

To simplify calculations, all materials were assumed to be linearly elastic, homogenous, and isotropic. The elastic modulus, Poisson’s ratio, and yield strength for each material are summarized in Table 3 [1, 4, 23, 27].

**Table 3:** Summary of the material properties used for the finite element analysis

	Elastic Modulus (MPa)	Poisson Ratio	Yield strenght	Reference
Ti Grade 5	116,000 MPa	0.31	790 MPa	Mommaerts, 2019 Carnicero, 2021 [1, 4]
Ti Grade 4	110,000 MPa	0.34	485 MPa	Vaidyanathan AK. 2022 [23]
PEEK	3800 MPa	0.37	108 MPa	Altiparmak N. et al., 2023 [26]
Cobalt-chromium	260,000 MPa	0.3	450 MPa	Gümrükçü, 2019 [27]
Acrylic resin	3000 MPa	0.35	70 MPa	Gümrükçü, 2019 [27]
Cortical bone	13,700	0.3	114 MPa	Gümrükçü, 2019 [27] and Hernandez CJ. 2001 [24]
Cancellous bone	1370	0.3	60 MPa	Gümrükçü, 2019 [27] and Keaveny TM. 2001 [25]

*Co-Cr* Cobalt-chromium, *MPa* Megapascal, *PEEK* Polyetheretherketone, *Ti* Titanium

**Fig. 3:**
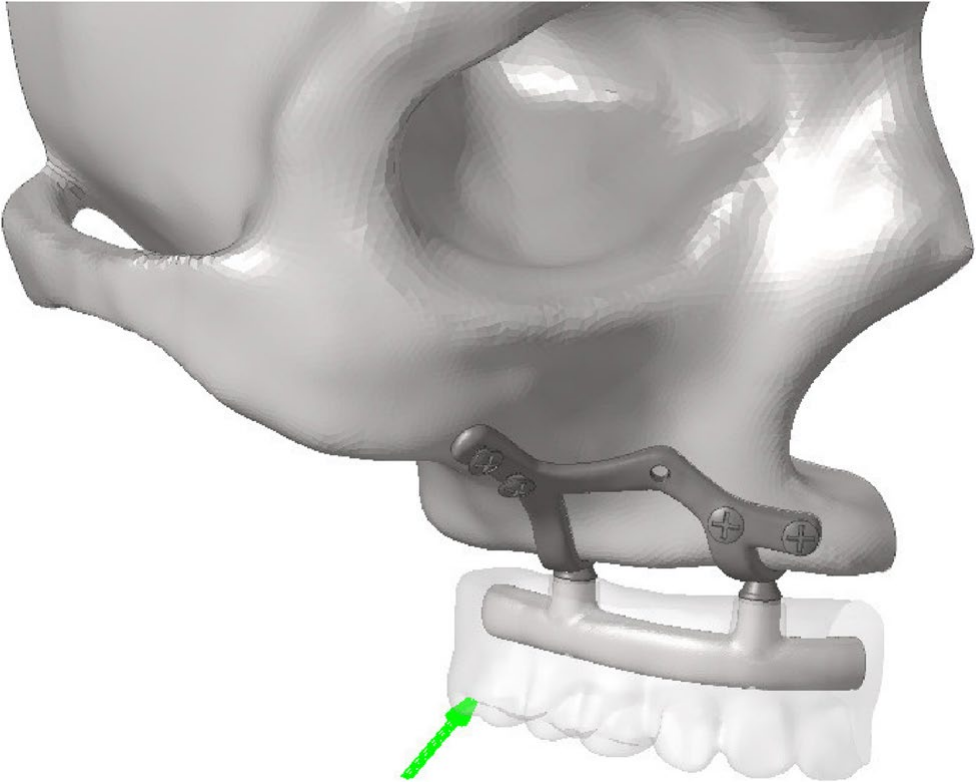
Direction of applied force

A total of eight linear static analyses were performed under the specified forces and boundary conditions, incorporating four different AMSJI designs, two different framework materials, and a single loading condition. A 200 N oblique force at a 45° angle was applied to the most distal part of the prosthesis (Fig. 3). The analysis evaluated Von Mises stresses in the subperiosteal implant, fixation screws, and framework, as well as maximum/minimum principal stresses in cortical and spongious bone along with the prosthesis displacement.

The Von Mises stress value was used to analyze material behavior, as it is appropriate for predicting failure in ductile materials such as titanium, PEEK, and Co-Cr (Fig. 1C and F) [23]. Higher Von Mises stress values indicate an increased risk of failure [2, 28–30].

## Results

**Fig. 4:**
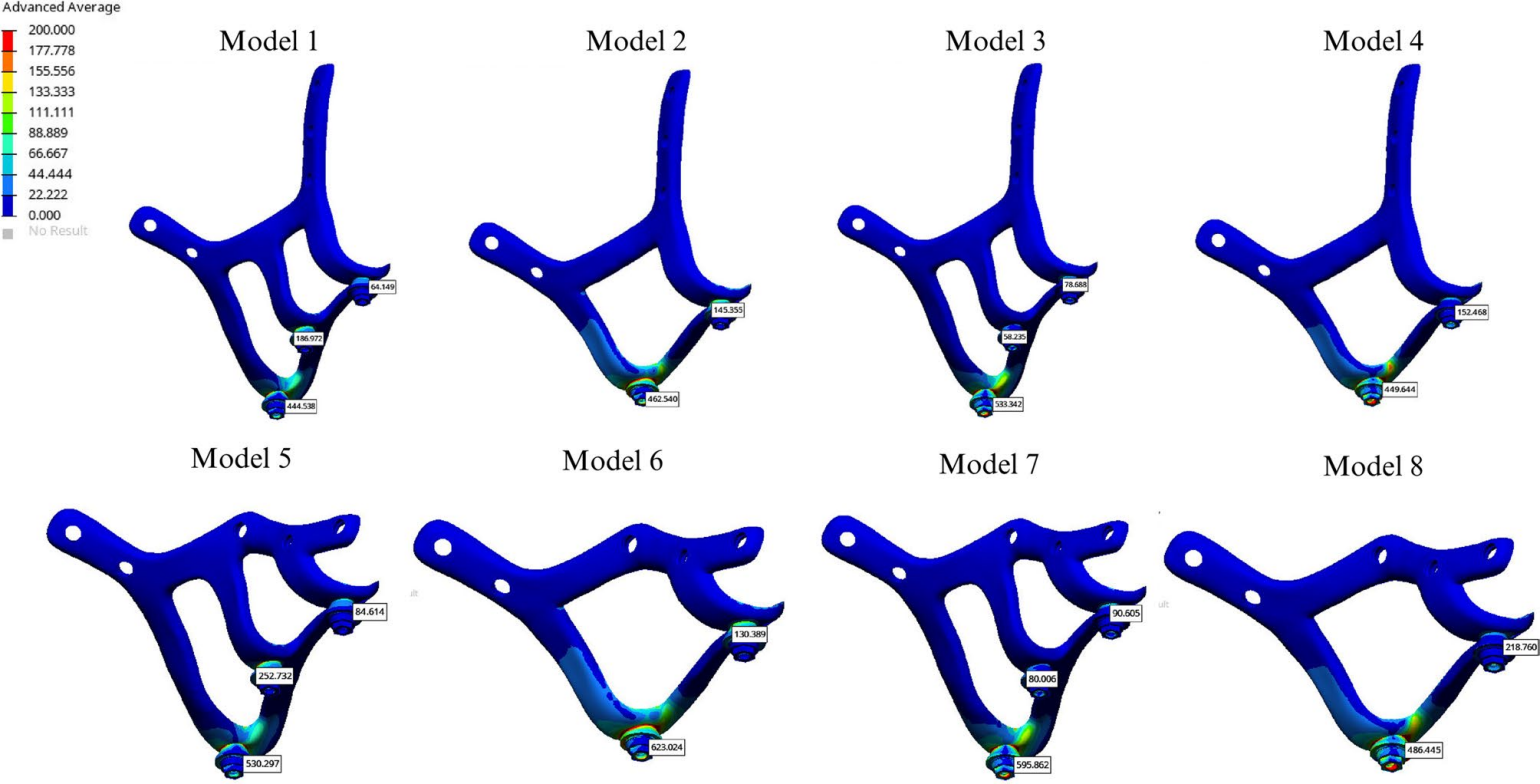
Von Mises values on subperiosteal implant

The lowest stress value observed in the implant was 444.538 MPa in the Co-Cr group without cantilever extension, using the I-shaped design. The second lowest stress value, 449.644 MPa, was recorded in the PEEK group with a cantilever, also utilizing the I-shaped design (Fig. 4).

Among all groups, the highest stress value was found in the cantilevered Co-Cr group with the Y-shaped design (623.024 MPa). For the I-shaped design, the highest implant stress was found in the PEEK group without a cantilever extension (533.342 MPa) (Table 4). Stress levels were significantly lower in Groups 4 and 8 (449.644 and 486.445 MPa, respectively), whereas Groups 3 and 7 exhibited higher stress values (533.342 and 596.862 MPa, respectively) (Fig. 4).

**Table 4:** All stress values are given in megapascal unit

Group	Von Mises AMSJI	Von Mises Framework	Von Mises Fixation screw	Minimum Principal Cortical Bone	Minimum Principal Trabecular Bone	Maximum Principal Cortical Bone	Maximum Principal Trabecular Bone	Prosthetic Displacement value
1	444.538	131.162	12.799	20.325	2.389	9.541	0.890	0.163
2	462.540	291.856	17.493	32.328	4.036	12.280	0.994	0.245
3	533.342	71.029	10.241	22.330	2.741	14.036	1.645	0.362
4	449.644	103.635	34.468	34.350	4.259	10.069	1.026	0.727
5	530.297	132.845	10.142	20.846	2.395	9.979	0.991	0.163
6	623.024	292.043	17.882	29.799	3.656	12.885	0.958	0.245
7	595.862	71.249	10.094	22.891	2.751	14.070	1.944	0.362
8	486.445	103.666	35.056	32.629	3.862	10.356	1.096	0.727

*AMSJI* Additively manufactured subperiosteal jaw implant

**Fig. 5:**
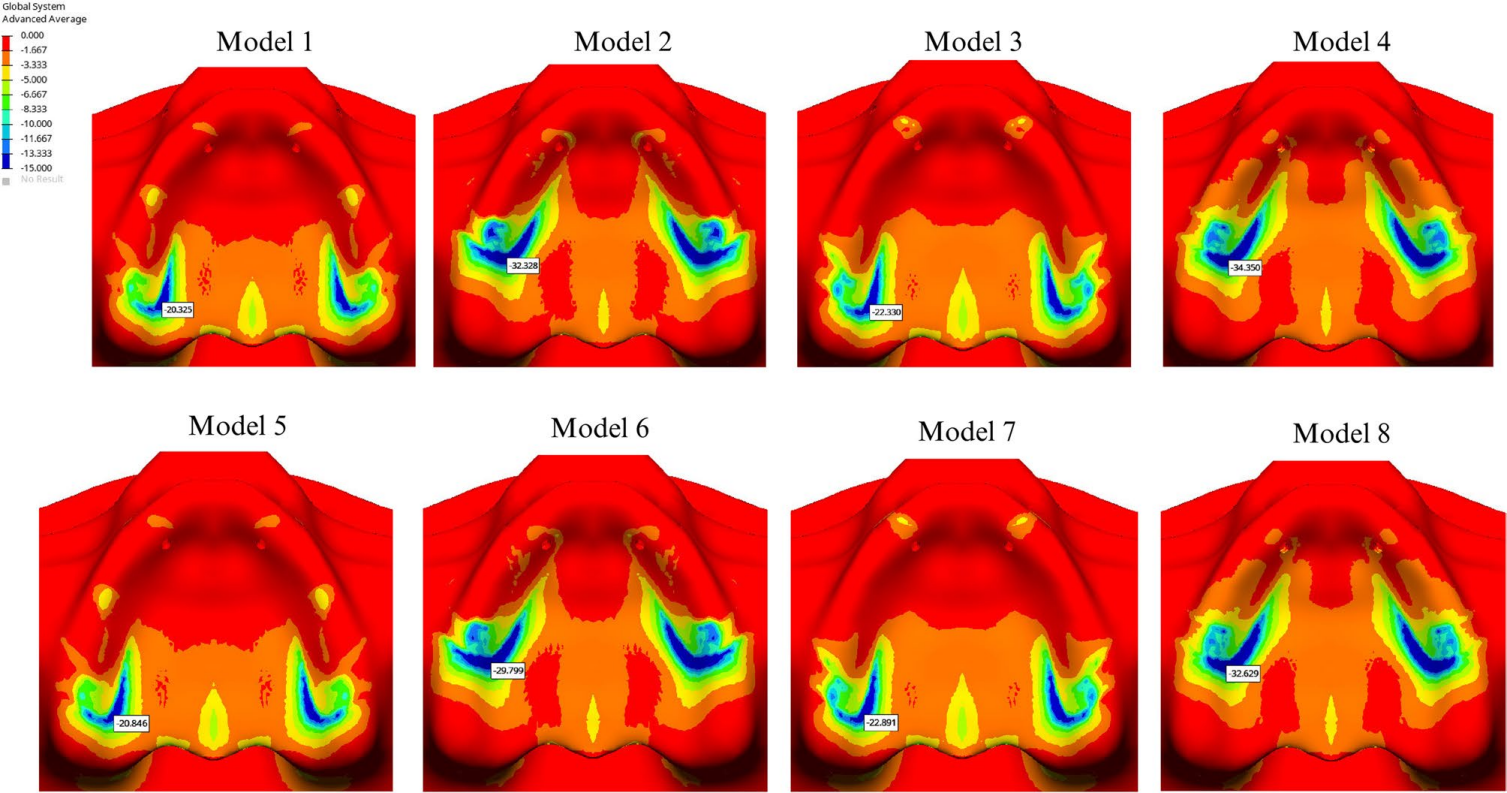
Highest compressive stress in cortical bone

**Fig. 6:**
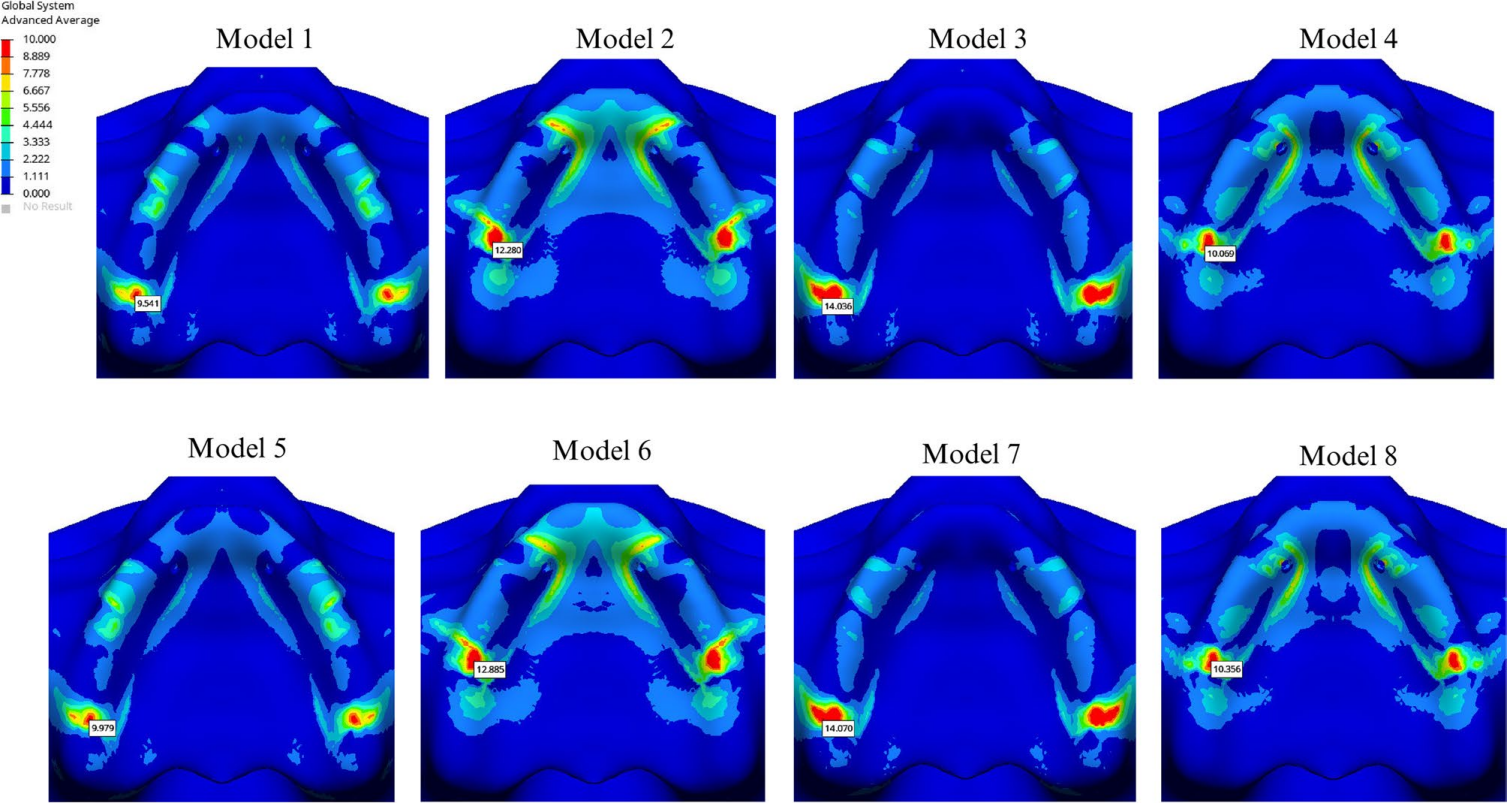
Highest compressive stress in trabecular bone

In groups using PEEK, stress values in the framework were lower compared to those using Co-Cr. The presence of a cantilever increased stress in the framework material, regardless of the design. In cantilevered groups, a PEEK framework helped reduce implant stress, whereas in Co-Cr groups, all materials exhibited increased stress levels in cantilevered configurations. The highest stress value in the implant screws was recorded at the 5th screw, located on the palatal side. Additionally, the Y-shaped design significantly increased stress levels at the 1 st and 2nd screws compared to the I-shaped design (Table 4). The highest compressive stress values in the cortical and trabecular bone were observed in the palatal region of the abutment closest to the applied force and around the 5th screw, aligning with the stress concentration areas in the implant and screws. The Y-shaped design helped reduce stress levels in the cortical bone on the palatal side of the most distal abutment in the cantilevered design. The highest compressive stress in the cortical bone (34.350 MPa) was observed around the most distal abutment in the I-shaped design within the cantilevered PEEK group (Fig. 5). Additionally, the highest cortical bone compressive stress (3.448 MPa) around the 5th screw occurred in the Y-shaped design, in conjunction with a cantilever extension and a PEEK framework. In all cases, minimum principal stress values were higher in the cortical bone than in the cancellous bone. Comparatively, compressive stress levels in both cortical and trabecular bone were higher in the PEEK group than those in the Co-Cr group (Figs. 5 and 6).

**Fig. 7:**
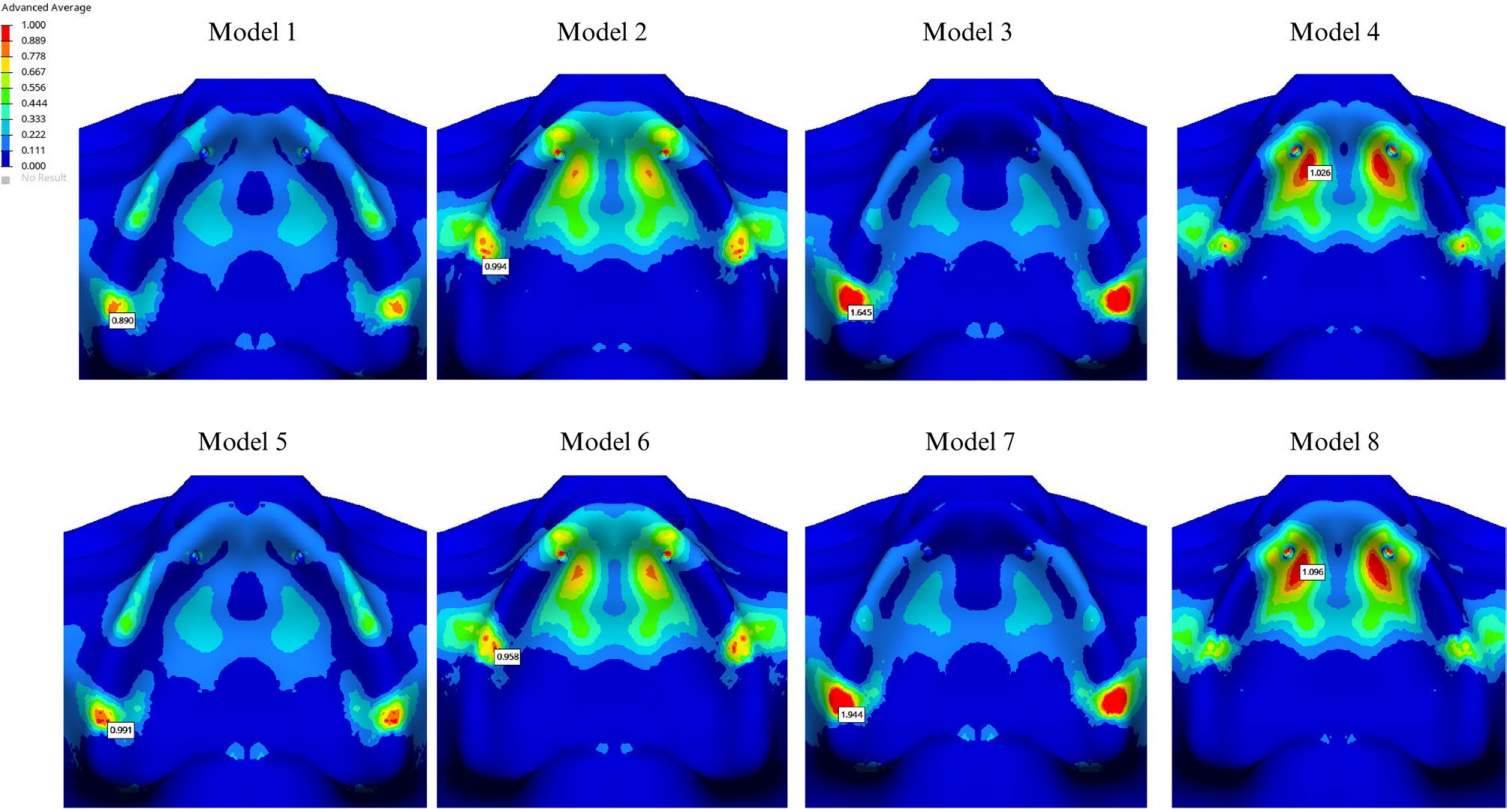
Highest tensile stress in cortical bone

**Fig. 8:**
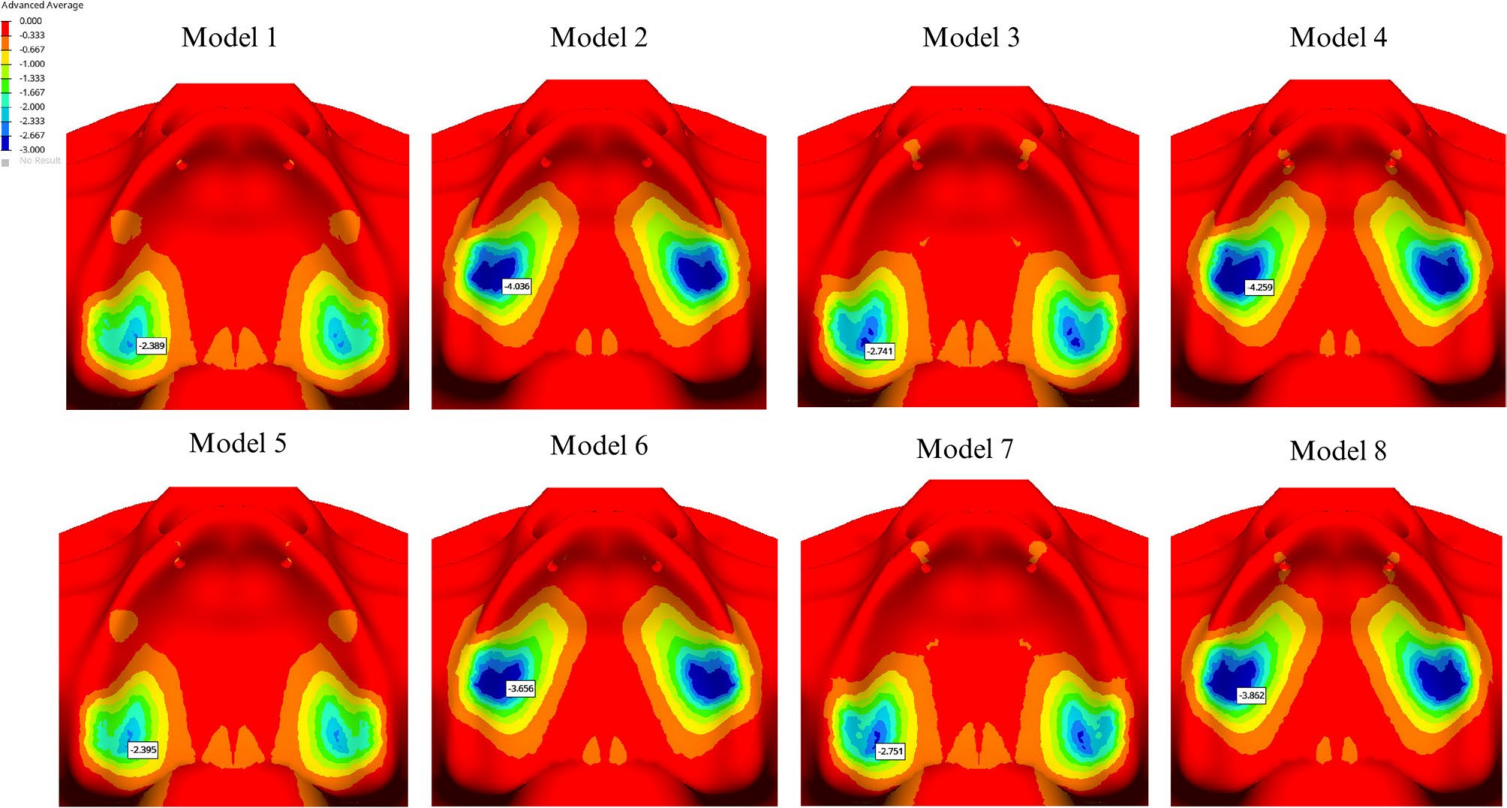
Highest tensile stress in trabecular bone

The highest stress values in the cortical bone were detected on the buccal side of the abutment closest to the applied force. The presence of a cantilever, regardless of the framework type, led to increased stress around the screws. In the cantilever-free group, tensile stress values were higher in the PEEK group for both cortical bone (14.070 MPa) and trabecular bone (1.645 MPa) compared to those in the Co-Cr group (Figs. 7 and 8). Conversely, in cantilevered groups, tensile stress values around the abutment were higher in the Co-Cr group than in the PEEK group. The highest stress values in the cortical and trabecular bone were observed around the 5th screw (7.934 MPa and 0.986 MPa, respectively).

**Fig. 9:**
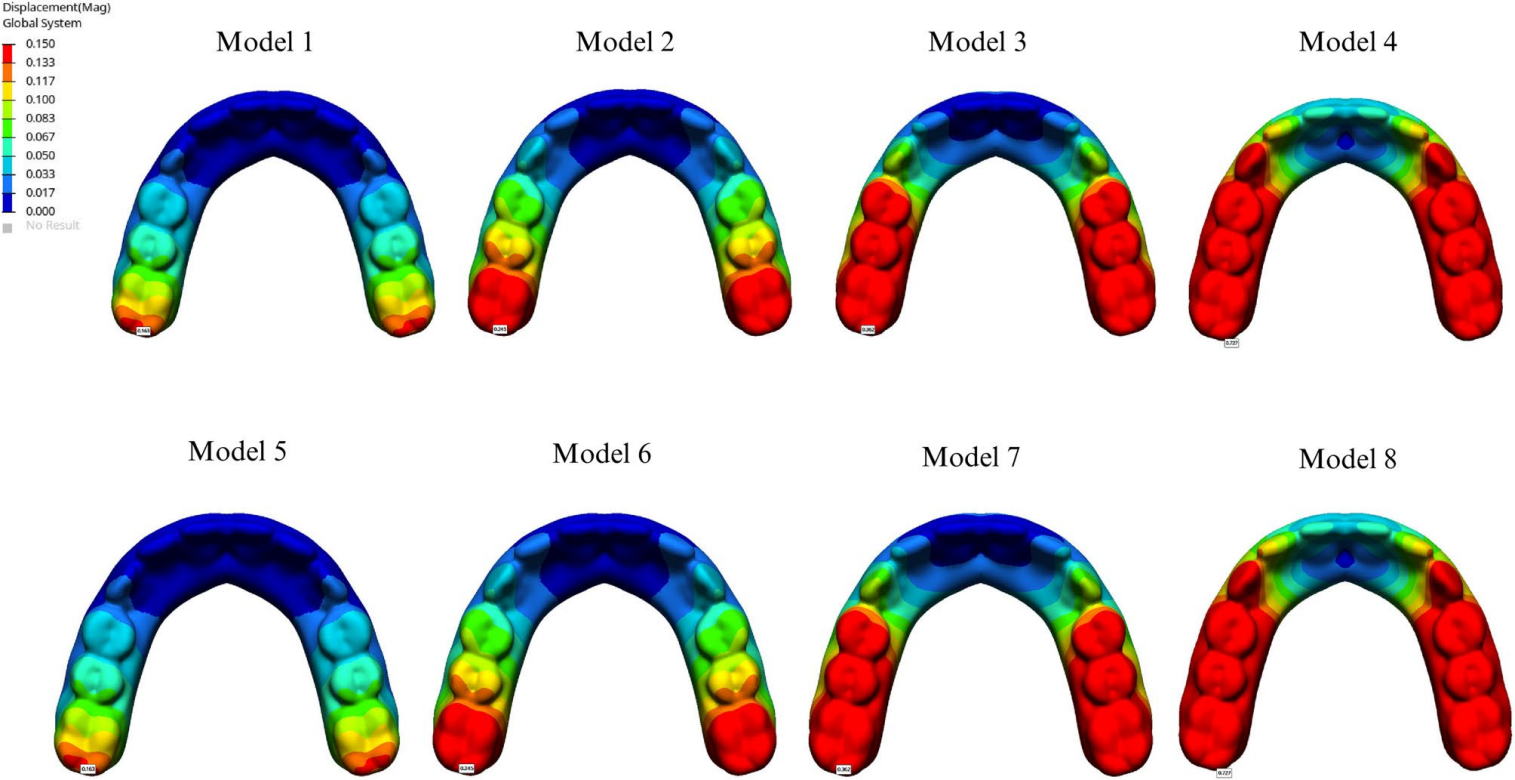
Displacement values

The maximum displacement values for each group are summarized in Table 4. Analysis of prosthetic displacement showed no significant difference between the I- and Y-shaped designs. However, displacement was greater in cantilevered groups than in non-cantilevered groups, with the PEEK material exhibiting higher displacement than the Co-Cr material (Fig. 9).

## Discussion

Advancements in implant research, particularly in material science and biological interactions, have significantly improved the design of subperiosteal implants. The integration of technologies such as CT, intraoral and extraoral scanners, and CAD/CAM software has revolutionized the production of these implants through additive manufacturing. These custom-designed implants, precisely tailored to a patient’s bone structure, such as subperiosteal implants, provide a valuable alternative in cases where conventional treatments are not viable [31]. These innovations have led to more effective, personalized treatments, offering enhanced precision and significantly improved outcomes in implantology. Within this context, the study established eight homogeneous and isotropic groups by combining four distinct implant designs with two different infrastructure materials. These groups underwent stress analysis to explore potential solutions to common clinical challenges. The findings of this study are expected to contribute to the continued advancement and refinement of subperiosteal implants by addressing real-world challenges and enhancing the overall success and efficacy of implant-based treatments. Co-Cr exhibits high elastic modulus and endurance, making it significantly resistant to applied forces [32, 33]. These properties generally facilitate the uniform distribution of force. This characteristic contributes to a more uniform force distribution, which explains why groups using Co-Cr infrastructures exhibited reduced stress concentration in the subperiosteal implant. Additionally, this factor may help mitigate the formation of microcracks or damage in the surrounding bone. In cases of proprioceptive loss, particularly in patients with bruxism who exert higher occlusal forces, using an infrastructure material with a high elastic modulus can help prevent plastic deformation and structural failure of the AMSJI.

In contrast, PEEK’s more flexible nature allows it to absorb stresses, thereby reducing stress within the framework [34]. However, studies have shown that under oblique loading around posterior implants, PEEK generates higher stress levels in the cortical bone [14]. Framework materials with lower elastic modulus, such as PEEK, tend to bend more under functional loads in the prosthesis, which consequently increases stress transmission to the implant. Due to its low elastic modulus, PEEK transmits a greater portion of the applied force to the underlying structures compared to Co-Cr [35, 36].

High-stress concentrations of approximately 13–14 MPa observed in the trabecular bone, consider critical as they may contribute to excessive loading and potential bone resorption [37]. The study’s findings align with previous research and indicate that PEEK may be a promising option as a prosthetic framework. Although this study recorded a maximum principal stress of 1.944 MPa in the trabecular bone, concerns remain regarding the long-term effects on the subperiosteal implant complex and surrounding bone. The forces transmitted by PEEK material to the underlying structures could lead to increased stress on both the implant and bone over time. Further long-term validation of this material would be beneficial.

The piriform aperture frame wings should be positioned at a distance from the crest apex while maintaining adequate separation from the nasolacrimal duct ostium and the head of the inferior turbinate [1]. If this approach is not feasible due to anatomical constraints or bone deficiencies, a design modification in the lateroinferior region of the nasal base may be considered to optimize bone utilization. Findings from this study indicate that the Y-shaped wing design led to increased stress levels on both the implant and surrounding bone across all groups. Although all stress values remained within physiological limits, the I-shaped wing design is recommended as the preferred option to minimize stress-related failure rates in subperiosteal implants. However, in cases with anatomical constraints, the Y-shaped design may serve as a suitable alternative.

To improve stress distribution, the use of a prosthesis without a cantilever is recommended. A study by Horita et al. found that non-cantilevered prostheses exhibited a 45.3–52.5% reduction in peak compressive stresses during loading compared to cantilevered prosthesis designs after immediate implant placement [21]. Consistent with these findings, this study also identified the presence of a cantilever as a factor that increases stress. In a comparison of PEEK and Co-Cr frameworks with varying cantilever lengths, the least deformation was observed in the non-cantilevered PEEK framework, whereas the highest deformation occurred in the PEEK framework with a cantilevered design [5]. Similarly, this study found that in cantilevered configurations, PEEK frameworks reduced load distribution due to their force-dissipating properties. The material’s elastic modulus and distinct force transmission characteristics resulted in increased deformation, which, in turn, reduced the force transmitted to the implant and underlying bone. The greater prosthetic displacement observed in the PEEK with the cantilevered group further supports this conclusion. The results suggest that in the presence of a cantilever, selecting an appropriate framework material is as important as the implant design in minimizing stress on the subperiosteal implant.

The connection points where the implant’s structural struts meet the abutments could represent a weak spot. In Mommaerts’ studies, the highest stress values were observed at the connection between the most distal abutment and its supporting arm, which is particularly sensitive to bending forces; this region was identified as the most critical area within the entire system [1, 22]. Similarly, this study recorded the highest stress levels in the most distal abutment of the system, directly subjected to the applied load, and in the underlying cortical bone.

Since the main structure of the subperiosteal implant rests on the alveolus, cases classified as borderline Cawood and Howell Class IV or V involve progressive residual ridge resorption. In this unsupported region, the risk of arm exposure increases, potentially leading to failure in the most critical stress-bearing area.

To address this, the abutment implant junction, which bears the highest stress load within the system, should be designed with maximum thickness. Furthermore, positioning the implant arms in areas less susceptible to resorption or securing them in more stable regions created through osteotomy at the crest top may significantly reduce the risk of failure. Additionally, the use of a rigid framework material that promotes homogeneous force distribution may enhance the overall mechanical stability of the implant complex under excessive loads.

Consistent with previous studies, this research found that compressive and tensile stress values were higher in the cortical bone compared to the trabecular bone, suggesting that the cortical bone assumes a protective role in stress distribution, helping reduce the overall force applied to the underlying structures [38–40].

In this study, stress distribution across the subperiosteal implant complex was assessed considering the application site and direction of the applied force. Predictably, the stress was distributed across the entire implant structure, with localized variations observed around specific screws. Where an oblique force was applied in the buccopalatal direction, the highest stress concentration consistently occurred at the 5th screw, likely due to its positioning, orientation, and length. Additionally, compared to the I-shaped design, the Y-shaped design increased stress on the 1 st and 2nd screws while effectively reducing stress on the 5th screw on the palatal side. The AMSJI concept relies primarily on mechanical retention through screw fixation rather than osteointegration for stability. Based on these findings, the strategic placement, angulation, and length of screws in AMSJI designs are vital factors in effectively minimizing long-term stress accumulation.

The study has certain limitations. FEA, while effective in evaluating stress distribution and material behavior, does not fully replicate intraoral conditions, limiting the ability to make direct clinical generalizations. Furthermore, the dynamic nature of living tissues and biological interactions cannot be accurately simulated. Moreover, the modeling process excluded the mucosa, assuming its influence on peri-implant bone stress distribution to be minimal. Notably, the behavior of the PEEK framework across different cantilever lengths was not explored. Finally, in all scenarios, the applied force was considered to be oblique; however, real-life occlusal forces vary in magnitude and direction with each cycle, which could influence fatigue resistance and long-term outcomes.

## Conclusion

Across all scenarios, the most optimal design regarding stress distribution was achieved with the I-shaped design without the cantilever, using a Co-Cr framework material. If a Y-shaped wing design is required, avoiding cantilever extensions and selecting a more rigid infrastructure material may be effective in optimizing stress distribution on the implant and surrounding bone. The stress analysis results suggest that PEEK offers potential benefits in configurations, as it helps reduce stress in the framework, bone, and implant. However, uncertainties remain regarding the long-term impact of PEEK’s flexibility on implant stability and, consequently, on the tolerance of surrounding tissues. Given the lack of definitive data on the long-term performance of PEEK, these findings should be further validated through long-term clinical trials.

## Data Availability

No datasets were generated or analysed during the current study.

## Abbreviations

AMSJI: Additively manufactured subperiosteal jaw implant
CT: Computed tomography
CAD/CAM: Computer-aided design and manufacturing
Co-cr: Cobalt-chromium
Corp: Corporation
DICOM: Digital Imaging and Communications in Medicine
ECC: Error-Correcting Code
FEA: Finite element analysis
Fig: Figure
GB: Gigabyte
GHz: Gigahertz
Inc: Incorporated
LS-DYNA: Livermore Software for Dynamic Nonlinear Analysis
MPa: Megapascal
N: Newton
Peek: Polyetheretherketone
STL: Stereolithography
VR: Virtual Reality
3D: Three-dimensional
